# Statement of clarification

**DOI:** 10.1007/s13244-012-0198-4

**Published:** 2012-10-31

**Authors:** European Society of Radiology (ESR), European Association of Nuclear Medicine (EANM)

**Affiliations:** 1 European Society of Radiology (ESR), Neutorgasse 9/2, AT-1010 Vienna, Austria; 2 European Association of Nuclear Medicine (EANM), Hollandstrasse 14/Mezzanine, AT-1020 Vienna, Austria

Since its publication by the European Society of Radiology (ESR) and the European Association of Nuclear Medicine (EANM), the Multimodality Imaging Training Curriculum has given rise to numerous discussions regarding its aims and applicability [1–4].

Both societies therefore wish to clarify specifically the purpose and scope of the document. The aim of the Multimodality Imaging Training Curriculum as a joint European initiative is to advance the quality of performance and reporting of hybrid imaging by defining the scope of training for medical specialists in hybrid imaging. It outlines skills and knowledge requirements in nuclear medicine for those whose training background is in radiology and in radiology for those whose training background is in nuclear medicine.

This training curriculum is intended to relate solely to the training of specialists in hybrid imaging from both radiology and nuclear medicine with the purpose of ensuring optimal use of this complex methodology for patient care. It by no means encompasses full training and/or qualification in imaging modalities unrelated to hybrid imaging techniques.

## References

[1] European Association of Nuclear Medicine (EANM), European Society of Radiology (ESR) (2012). Multimodality imaging training curriculum—parts II and III. Insights Imaging.

[2] European Association of Nuclear Medicine (EANM), European Society of Radiology (ESR) (2012). Multimodality imaging training curriculum—parts II and III. Eur J Nucl Med Mol Imaging.

[3] European Society of Radiology (ESR), European Association of Nuclear Medicine (EANM) (2011). Multimodality imaging training curriculum—general recommendations. Insights Imaging.

[4] European Association of Nuclear Medicine (EANM), European Society of Radiology (ESR) (2011). Multimodality imaging training curriculum—general recommendations. Eur J Nucl Med Mol Imaging.

